# SiC formation for a solar cell passivation layer using an RF magnetron co-sputtering system

**DOI:** 10.1186/1556-276X-7-22

**Published:** 2012-01-05

**Authors:** Yeun-Ho Joung, Hyun Il Kang, Jung Hyun Kim, Hae-Seok Lee, Jaehyung Lee, Won Seok Choi

**Affiliations:** 1 School of Electrical Engineering, Hanbat National University, Daejeon 305-719, Republic of Korea; 2 Department of Applied Materials Engineering, Hanbat National University, Daejeon 305-719, Republic of Korea; 3 Solar Cell R&D Center, Shinsung Solar Energy Co., Ltd., Seongnam 463-420, Republic of Korea; 4 School of Information and Computer Engineering, Sungkyunkwan University, Suwon 440-746, Republic of Korea

**Keywords:** a-Si_1-x_C_x_, passivation layer, RF magnetron co-sputtering system, carrier lifetime, solar cell

## Abstract

In this paper, we describe a method of amorphous silicon carbide film formation for a solar cell passivation layer. The film was deposited on p-type silicon (100) and glass substrates by an RF magnetron co-sputtering system using a Si target and a C target at a room-temperature condition. Several different SiC [Si_1-x_C_x_] film compositions were achieved by controlling the Si target power with a fixed C target power at 150 W. Then, structural, optical, and electrical properties of the Si_1-x_C_x _films were studied. The structural properties were investigated by transmission electron microscopy and secondary ion mass spectrometry. The optical properties were achieved by UV-visible spectroscopy and ellipsometry. The performance of Si_1-x_C_x _passivation was explored by carrier lifetime measurement.

## Introduction

Semiconductor technology or microelectronics including solar cells has been adopted to form micro- or nano-sized state-of-the-art structures which can reduce system size, improve its performance, achieve lower system cost, and so on [1-3]. Generally, a semiconductor structure or system is manufactured by a combination of additive (film deposition) and subtractive (etching) processes. These days, many research have tried to utilize the microelectronic technology (especially in the deposition of thin film layers) to get more efficient and cost-effective solar cells [4,5].

Amorphous silicon-based thin film layers (SiO_2_, SiN, a-SiC:H, and so on) for antireflection coatings, diffusion barriers, passivation layers, and silicon bulk materials have been broadly researched in the solar cell industry. Among the film layers, SiO_2 _and SiN passivation layers have highly attracted to fabricate high-efficiency silicon solar cells. However, they have negative demerits such as the need for a high-temperature process, difficulty in the photolithography process, and poor thermal stability [6-8]. Hydrogenated amorphous silicon carbide [a-SiC:H] has been studied for solar cell passivation layers due to its wide bandgap, excellent coefficient of thermal expansion that matches with silicon wafers, relatively good thermal and mechanical stabilities, superior cost-of-ownership compared to other materials, and so on. The formation or deposition of the a-SiC:H film has mainly been done by plasma-enhanced chemical vapor deposition [9,10]. However, the thermal stability of the hydrogen-containing film is degraded during a post-high-temperature firing process. To avoid the hydrogen molecule's void generation, the authors have proposed an a-SiC deposition method by radio frequency [RF] sputtering which was performed by a single silicon-carbide composite target in an argon environment [10]. In this paper, we introduce a deposition method of amorphous silicon carbide [a-Si_1-x_C_x_] which was done by RF magnetron co-sputtering. The method can utilize multiple targets (in this paper, Si and C) simultaneously in order to deposit complex compositional coatings. The compositional ratio can be controlled by the differences in sputtering yield, the relative ability of the materials to stick to the substrate, the deposition temperature, and the relevant percent of sputtered elements to reach the substrate without being scattered from the plasma. In this paper, we investigate several properties of the compositional films by controlling the Si target's RF power. Film thickness was measured using a field-emission scanning electron microscope [FE-SEM]. Reflective index of the film was obtained using an ellipsometer, and carrier lifetime of the film on the doped p-type silicon wafer was obtained by a silicon wafer lifetime tester.

## Experimental details

**Figure 1:**
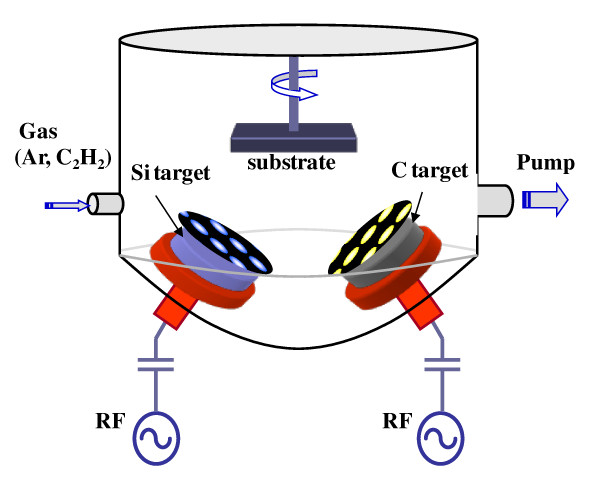
**A schematic diagram of the RF magnetron co-sputtering system**.

**Figure 2:**
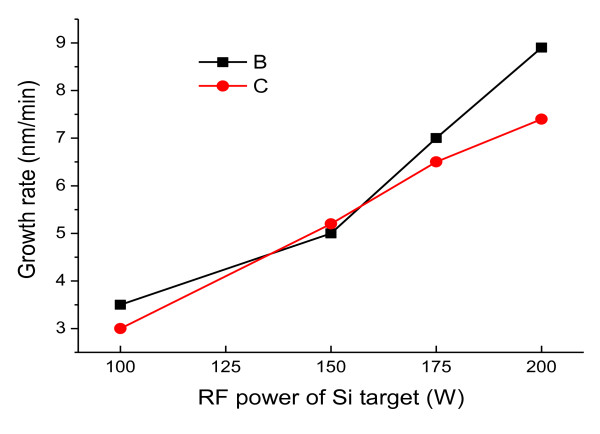
**Deposition rate of a-Si_1-x_C_x _passivation layer as a function of the Si target's RF power**.

The a-Si_1-x_C_x _passivation layer was deposited on 2 × 7-cm glass substrates and 4-inch p-type silicon (100) wafers using an RF magnetron co-sputtering system. Figure 1 shows the schematic diagram of our RF magnetron co-sputtering system. Before the RF plasma process, the substrates were cleaned in trichloroethylene, acetone, methanol, and distilled water for 10 min. For the silicon wafers, an acid treatment was added for 45 s. Pure (99.9%), 4-inch Si and C targets were installed to achieve a high-quality a-Si_1-x_C_x _passivation layer. The sputtering chamber was vacuumed up to the base pressure of 1 × 10^-5 ^Torr using a turbomolecular pump. A highly pure (99.9999%) argon environment was established for the deposition with a flow of 40 sccm. Then, the Si and C targets were pre-sputtered to clean the target surface and the chamber for 10 min. The target-to-substrate distance and the substrate rotation speed were fixed for all depositions as 6 cm and 1, 700 rph, respectively. In this work, to check the effect of the Si-to-C ratio on the a-Si_1-x_C_x _passivation layers, the RF power of the C target was fixed at 150 W, and several different RF powers (100, 150, 175, and 200 W) of the Si target were applied to. However, the thicknesses of all a-Si_1-x_C_x _passivation layers were kept constant at 100 nm by controlling the deposition rate. The deposition rates with the different RF powers of the Si target were summarized in Figure 2, and the detailed experimental parameters were summarized in Table 1.

**Table 1:** Deposition conditions of the a-Si_1-x_C_x _passivation layer

Deposition parameters	Conditions
Substrate	Glass and Si substrates
Base pressure	0.01 mTorr
Working pressure	3 mTorr
RF power	C target, 150 W
	Si target, 100; 150; 175; and 200 W
Target-to-substrate distance	6 cm
Rotation speed	1, 700 rph
Target	4-inch Si and C
Sputtering gas	Ar, 40 sccm
Substrate temperature	RT

RF, radio frequency; RT, room temperature.

The deposited a-Si_1-x_C_x _passivation layer's thickness and crystal structure were measured using an FE-SEM (S-4800, Hitachi, Tokyo, Japan) and a transmission electron microscope [TEM] (JEM-2100F, JEOL, Seoul, South Korea), respectively. Optical properties of the transmittance and bandgap were measured by UV-visible spectroscopy (S-3100, Scinco, Seoul, South Korea), the refractive indexes were obtained using an ellipsometer (M2000D, Woollam, Uiwang-si, South Korea), and the electrical performance of the a-Si_1-x_C_x _passivation layer was analyzed by carrier lifetime measurement (WCT-120, Sinton Consulting Inc., Boulder, CO, USA).

## Results and discussion

**Figure 3:**
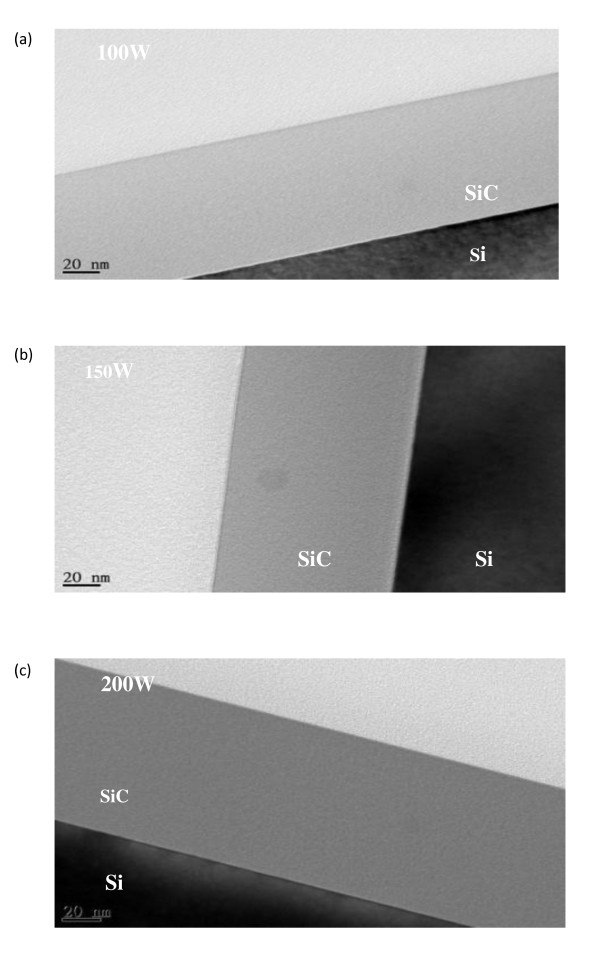
**TEM images**. At (**a**) 100-, (**b**) 150-, and (**c**) 200-W RF powers of the Si target.

As mentioned in the 'Experimental details' section, the film thickness was fixed at 100 nm. The thickness was measured with three different apparatuses, such as FE-SEM, TEM, and secondary ion mass spectrometer [SIMS]. Figure 3 shows cross-sectional bright field TEM images with a variation on the RF power of the silicon target. As the RF power of the Si target goes higher, the color of the Si1-xCx film gets darker due to the Si-rich compositional ratio of the film. It may indicate that the compositional ratio of the SiC is changed by the RF target power of the element.

**Figure 4:**
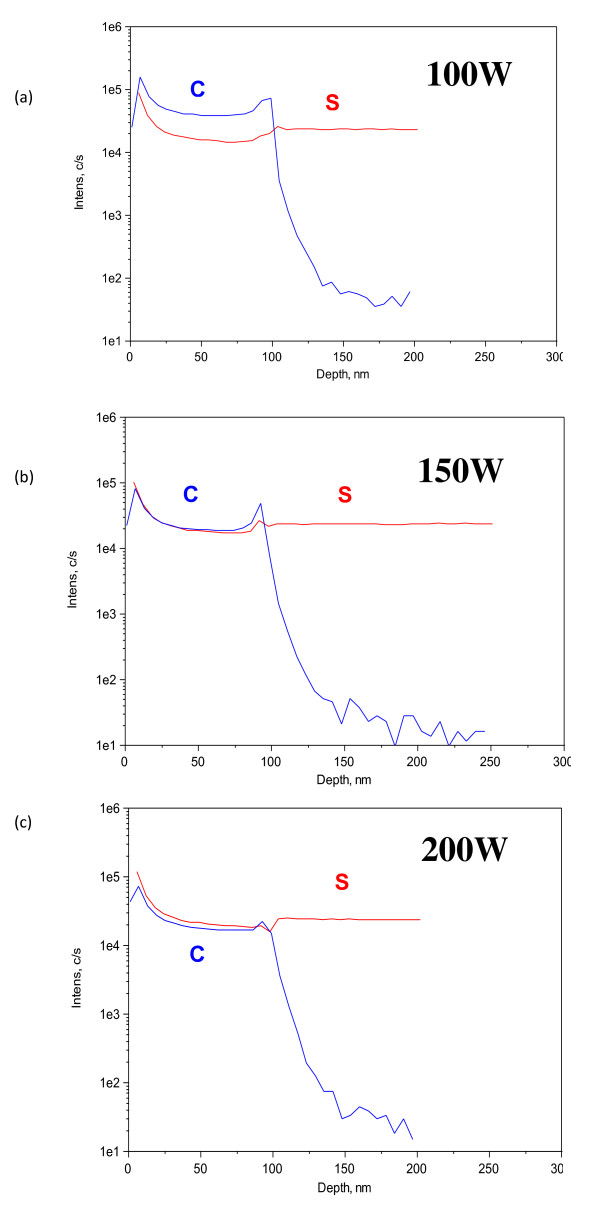
**Depth profiles as functions of the Si target's RF powers**. (**a**) 100, (**b**) 150, and (**c**) 200 W.

SIMS is widely used in the profile distribution of compositional elements along the deposited film depth by detecting the ionized element's mass. Figure 4 shows the depth profiles of Si and C along the deposited film. By increasing the RF power of the Si target, the mass intensity of the silicon particle gets higher than that of carbon. When we applied the same RF power on both targets, the compositional ratio of the elements seems to be 50:50 in mass intensity. When it goes deeper near the p-type silicon surface region, the carbon intensity is decreased abruptly. The falling point of the carbon intensity indicated the thickness of the a-Si1-xCx film with a sharp boundary between the film and the substrate.

**Figure 5:**
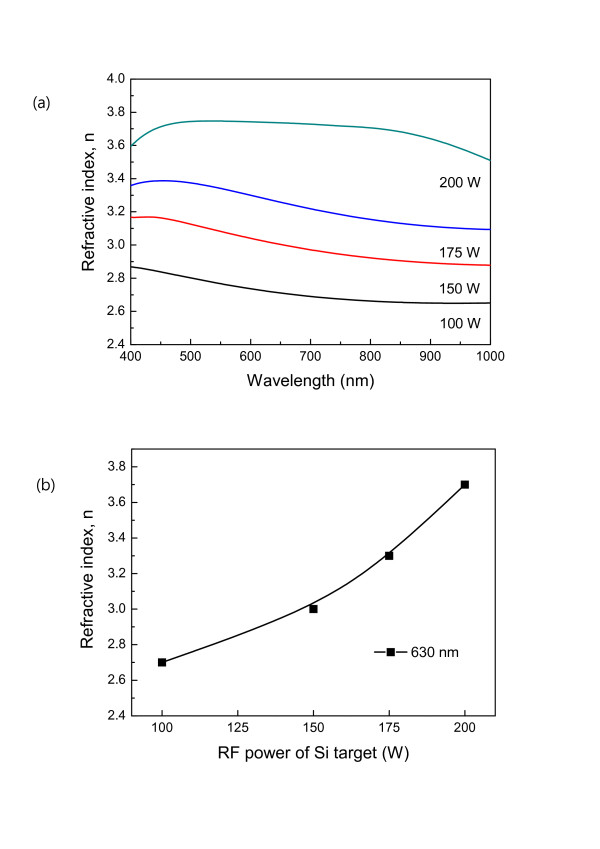
**Refractive index of a-Si_1-x_C_x _passivation layer as a function of the Si target's RF power**. (**a**) Refractive index, *n*. (**b**) One point of refractive index, *n *(630 nm).

Figure 5a shows the refractive index spectra of the a-Si_1-x_C_x _passivation films measured using an ellipsometer as a function of the Si target's RF power. The refractive indexes in the wavelength range of 400 to 1, 000 nm are increased when a higher RF power of the Si target is applied. At a 200-W RF power of the Si target, the refractive index value is highest all over the wavelength range. Generally, the refractive index of Si is higher than that of C (Si ≈ 3.49, C ≈ 2.41 at 630 nm). Therefore, when the RF power of the Si target goes up, more Si ions are deposited on the film. This Si-rich film makes a higher refractive layer. Figure 5b describes one point of refractive index at 630 nm which is a standard wavelength for optical properties in the visible wavelength regime and is a red-light wavelength. We obtained a refractive index of 2.7 at the 100-W RF power of the Si target. Then, by increasing the RF power of the Si target, the refractive index goes up, and at the 200-W RF power, we achieved the highest refractive index of 3.7. This result has a very similar trend with our previous experiment which was done with a single SiC composite target [5]. The previous result has a very narrow refractive index variation (3.2 to 3.4) with the change of a large RF power range (150 to 300 W), but co-sputtering method shows a relatively large refractive index variation (2.7 to 3.7). That is, we have more controllability of the refractive index selection for the passivation layer.

**Figure 6:**
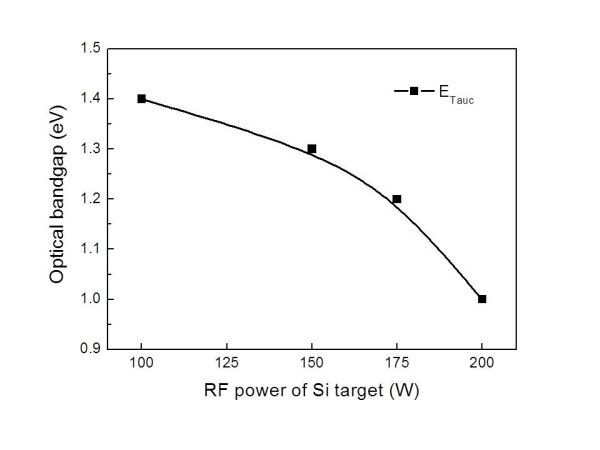
**Optical bandgap as a function of the Si target's RF power**.

The optical bandgaps were calculated from optical absorption measurement of the thin films which were deposited on the glass substrates. The optical absorptions were obtained from the intensity of the light measured by UV-visible spectroscopy [11]. Then, the optical bandgaps of the films were determined by the Tauc plot method which was performed by extrapolating the linear part of the absorption vs. photon energy (αhν)^1/2 ^curves [12,13]. Figure 6 shows the optical bandgap as a function of the RF power of the Si target. The absorption bandgaps are decreased from 1.4 to 0.9 eV as the RF power of the Si target is increasing. In general, the optical bandgap of carbon (≈5.5 eV at room temperature [RT]) has a much higher value than that of Si (≈1.11 eV at RT). When the Si target power is increasing, the film is changed to a Si-abundant layer. Therefore, it is reasonable that the higher RF power of the Si target brings a thin film with a lower optical bandgap.

**Figure 7:**
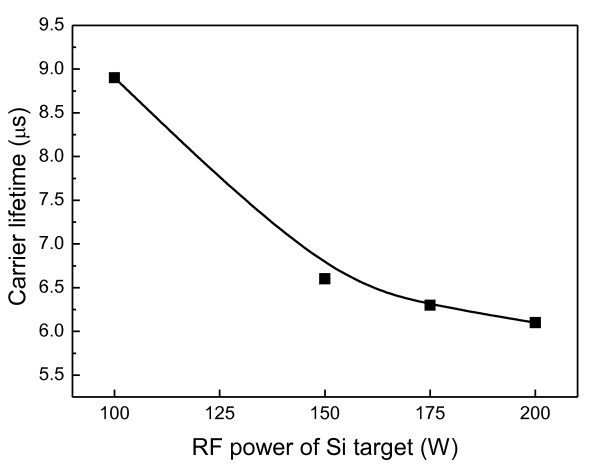
**Carrier lifetime of a-Si_1-x_C_x _passivation layer as a function of the Si target's RF power**.

Figure 7 shows the carrier lifetime of the a-SiC passivation layer measured by a silicon wafer lifetime tester with the function of Si target power. The carrier lifetime of the passivation layer deposited with a 100-W Si target power was 8.9 μs. Samnanta et al. reported that impurity in the semiconductor can degrade the lifetime by the creation of an electrical defect center or crystal imperfection [14]. Therefore, a carbon-rich thin layer can have more microdefects than a Si-rich film. In our study, the carrier lifetime was gradually decreased from 8.9 to 6.0 as the RF power of the Si target is increased from 100 to 200 W.

## Conclusions

We demonstrate a formation method of an a-Si_1-x_C_x _passivation layer for Si solar cells. The method was performed by RF magnetron co-sputtering with a Si target and a C target. The a-Si_1-x_C_x _passivation layer was deposited on p-type silicon (100) and glass substrates with the reaction of argon (Ar) gas. In this work, we have checked the effect of the Si-to-C ratio on the a-Si_1-x_C_x _passivation layers with variation on the RF powers of the Si target (100, 150, 175, and 200 W) and with fixation on the RF power of the C target (150 W). However, the thicknesses of all a-Si_1-x_C_x _passivation layers were kept constant at 100 nm. The SIMS profile showed that the film composition is changed with a variation on the RF power of the Si target. The refractive index analysis indicated that a Si-rich thin film has higher refractive index. We could obtain the highest refractive index of 3.7 with a 200-W RF power of the Si target. The optical bandgap analysis showed that the bandgap of the Tauc plot was observed to decrease gradually from 1.4 to 0.9 eV. The carrier lifetime analysis showed that the carrier lifetime of the a-Si_1-x_C_x _passivation layer was observed to decrease gradually from 8.9 to 6.0 μs as the carbon ratio in the film is decreased by changing the Si target power. Therefore, we could conclude that the RF magnetron co-sputtering method for SiC can deposit a thin film passivation layer for solar cells with various compositional ratios of Si and C.

## Competing interests

The authors declare that they have no competing interests.

## Authors' contributions

Y-HJ and WSC carried out the molecular genetic studies, participated in the sequence alignment, and drafted the manuscript. HIK and JHK carried out the immunoassay sample preparation. H-SL and JL participated in the sequence alignment. All authors read and approved the final manuscript.
